# Evaluation of Cosmetic and Dermatological Properties of Kombucha-Fermented Berry Leaf Extracts Considered to Be By-Products

**DOI:** 10.3390/molecules27072345

**Published:** 2022-04-06

**Authors:** Aleksandra Ziemlewska, Zofia Nizioł-Łukaszewska, Martyna Zagórska-Dziok, Tomasz Bujak, Magdalena Wójciak, Ireneusz Sowa

**Affiliations:** 1 Department of Technology of Cosmetic and Pharmaceutical Products, Medical College, University of Information Technology and Management in Rzeszow, Sucharskiego 2, 35-225 Rzeszow, Poland; zniziol@wsiz.edu.pl (Z.N.-Ł.); mzagorska@wsiz.edu.pl (M.Z.-D.); tbujak@wsiz.edu.pl (T.B.); 2 Department of Analytical Chemistry, Medical University of Lublin, Aleje Raclawickie 1, 20-059 Lublin, Poland; magdalenawojciak@umlub.pl (M.W.); i.sowa@umlub.pl (I.S.)

**Keywords:** kombucha, *Rubus fruticosus* L., *Vaccinum myrtillus* L., *Ribes nigrum* L., *Fragaria vesca* L, by-products, antioxidants, anti-aging properties, skin cells, skin hydrations

## Abstract

Leaves of *Rubus fruticosus* L., *Vaccinum myrtillus* L., *Ribes nigrum* L. and *Fragaria vesca* L. are considered agro-waste of the berry industry, but they can be a rich source of valuable bioactive compounds used in cosmetic industry. In this study, kombucha-fermented and non-fermented extracts were compared in terms of chemical composition and biological activity. Polyphenol compounds were identified by HPLC/DAD/ESI-MS. The antioxidant potential was analyzed by evaluating the scavenging of intracellular free radicals contained in keratinocytes and fibroblasts and by DPPH and ABTS assay, obtaining a higher radical scavenging capacity for the ferments, especially for *R. fruticosus* and *V. myrtillus* ferments. Assessment of the cytotoxicity on skin cell lines showed their positive effect on the viability of fibroblasts and keratinocytes (especially for the ferments after 10 days of fermentation). The potential anti-ageing properties were determined by their ability to inhibit the activity of metalloproteinases, obtaining almost 30% inhibition of collagenase and elastase in the case of fermented *V. myrtillus*. Moreover, when the samples were applied to the skin, the positive effect of ferments on skin hydration and pH was demonstrated, which indicates that kombucha berry leaf extracts may be an innovative cosmetic ingredient.

## 1. Introduction

The use of raw materials of plant origin in products intended for the care and treatment of various skin diseases is a common practice [1,2]. Flowers, fruits, leaves, roots, seeds, skins and plant herbs are very often used to obtain extracts used in the production of cosmetic and pharmaceutical preparations [3,4]. One commonly used raw material in cosmetology is berries [5], but very often, the leaves of these plants are thrown away and treated as waste. However, it is worth mentioning that they can be a rich source of valuable biologically active substances with a wide range of applications in the preparation of cosmetic formulas [6]. Due to the constantly growing demand for plant raw materials in the cosmetics industry, there is a need to verify the feasibility of using the potential of those parts of plants that were previously a by-product [7]. Taking into account economic factors and the sustainable management of plant materials, it is important to rationally manage both the raw materials and products themselves, as well as by-products and waste generated during the production process. Due to numerous reports in the literature showing the enormous potential and extraordinary biological properties of these wastes, the challenge is to find various extraction and processing techniques for these raw materials in order to obtain extracts or ferments rich in stable and multifunctional chemical compounds [4,5,6,7].

The valuable properties of berry fruits such as *Vaccinum myrtillus* L., *Ribes nigrum* L., *Rubus fruticosus* L. and *Fragaria vesca* L. have been known for a long time, which contributed to the fact that they were widely used in the cosmetics, food and pharmaceutical industries [5,8]. The leaves of these plants, which have so far been seen mainly as agro-waste, are also a rich source of valuable bioactive compounds, which contributes to a wide range of their biological activity [9]. Research shows that these leaves have strong antioxidant properties, thanks to which they have a positive effect on the condition of skin cells [6]. Scientific reports also indicate that *Ribes nigrum* L. leaves may have stronger antioxidant properties than fruit [10,11]. The extracts of these berry leaves have a positive effect on the metabolism and proliferation of keratinocytes and fibroblasts, which indicates the possibility of their use in preparations applied to the skin due to the lack of cytotoxic effect [6]. Moreover, the compounds found in the leaves of *Vaccinum myrtillus* L. mitigate the negative impact of solar radiation on fibroblasts, which may contribute to the improvement of the skin condition and reduce the photoaging process. What is more, they can also inhibit collagen degradation, which allows the skin to keep its youthful appearance [12,13]. The leaves of *Rubus fruticosus* L. and *Fragaria vesca* L. also have the ability to inhibit metalloproteinases responsible for the degradation of collagen fibers, which suggests that they can be used in anti-aging cosmetics [9,14,15].

Research is conducted all over the world to improve the processes of processing plant waste in order to obtain bioactive compounds with desirable biological properties [16]. The process that can significantly increase the content of bioactive compounds is fermentation by yeast and other microorganisms [16,17]. At present, ferments obtained with the use of a symbiotic culture of bacteria and yeast (SCOBY), which enable obtaining a large amount of compounds with a broad spectrum of activity, are gaining popularity [18]. Although initially SCOBY was primarily used to ferment tea leaves, more and more research is underway using alternative raw materials, as well as by-products, that can be fermented using this consortium of bacteria and yeast [19,20,21,22,23]. These works indicate that this fermentation leads to the production of extremely valuable chemical compounds; hence, the search for new plant materials that can undergo this type of fermentation is justified.

The aim of this study was to compare the properties of extracts and ferments obtained from the leaves of berries such as *Vaccinum myrtillus* L., *Ribes nigrum* L., *Rubus fruticosus* L. and *Fragaria vesca* L. The study determined the content of biologically active compounds, antioxidant properties and the level of reactive forms of oxygen in skin cells exposed to the tested extracts and ferments. Their cytotoxicity and the possibility of inhibiting enzymes responsible for the degradation of collagen and elastin fibers were also evaluated. Additionally, the influence of the obtained ferments on hydration, transepidermal water loss and skin pH was assessed.

## 2. Results and Discussion

### 2.1. Determination of Bioactive Compounds

Determination of polyphenol compounds (identification and quantification) in the *R. fruticosus* L., *R. nigrum* L., *F. vesca* L. and *V. myrtillus* L. leaves and their kombucha ferments was performed using HPLC/DAD/ESI-MS. Table 1 shows the following information about the detected polyphenolic compounds. Details of MS identification are presented in Tables S1–S4 in the Supplementary Material. The obtained profile was similar to those reported in the literature [24,25,26,27].

**Figure 1:**
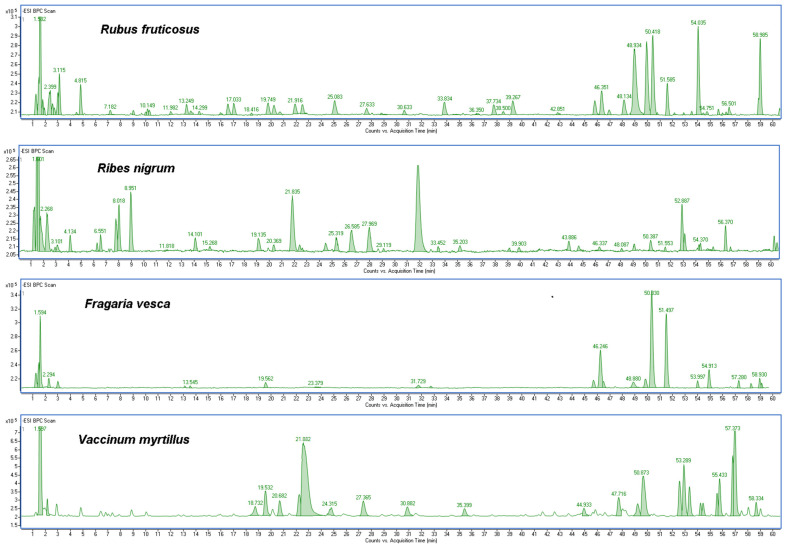
Representative BIC chromatograms for *R. fruticosus* L., *R. nigrum* L., *F. vesca* L., *V. myrtillus* L.

The results showed that berry leaves and their ferments are rich in polyphenol compounds, mainly phenolic acid—gallic and protocatechuic acid, hydroxycinnamates such as chlorogenic, neochlorogenic, and cryptochlorogenic acid; flavonoids—rutin, rutoside and quercetin derivatives: quercetin-3-*O*-galactoside, quercetin-3-*O*-glucuronide, quercetin malonyl glucoside or quercetin rhamnoside 3-*O*-glucoside; and derivatives of kaempferol—kaempferol-3-*O*-rutinoside and kaempferol-3-*O*-glucuronide. Additionally, ellagic acid and ellagic acid pentoside are found in the leaves of *R. fruticosus* L. and *F. vesca* L. The berry leaf chromatograms are presented in Figure 1. The obtained results, expressed in mg/mL of extract, are presented in Table 2. As demonstrated, the content of the determined compounds varied in the analyzed extracts. Fermented and non-fermented extracts were compared in terms of chemical composition. Typically, the contents of phenolic compounds and flavonoids in the samples tended to be higher for the ferments after 10 and 20 days of fermentation (F10, F20) than for the extracts. It was found that, for *R. fruticosus* L., ellagic acid was the most abundant compound (even 87.88 µg/mL ± 0.42 for F20) (for comparison, the content of this compound in the extract was 19.57 µg/mL ± 0.86). *V. myrtillus* L. has a high content of chlorogenic acid (1362.91 µg/mL ± 8.88 for F10 and 923.25 µg/mL ± 1.43 for the extract). In addition, significant differences in polyphenol content were observed between the extracts and their ferments for some of the compounds tested. For example, for *F. vesca* L., the gallic acid content for F10 and F20 is about 11 times higher than for the extract. It was also noted that, for many of the labeled compounds, there is no significant difference in their content when fermentation times (10 and 20 days) are compared.

The study showed that kombucha ferments had a higher content of active compounds as well as higher antioxidant activity than berry leaf extracts. Malbasa et al. noted that during the fermentation of black and green tea kombucha, in addition to phenolic compounds, other metabolites such as ascorbic acid and other organic acids are produced, which may also modify the antioxidant activity of kombucha [28]. Ivanišova et al. found significant amounts of phenolic compounds such as gallic acid, chlorogenic acid, protocatechuic acid, *p*-coumaric acid and ellagic acid in black tea kombucha. The authors also noted high values of total phenols, flavonoids and antioxidant capacity in kombucha, suggesting that complex phenolic compounds may be degraded to smaller molecules during fermentation [29]. Furthermore, the antioxidant capacity of kombucha is also affected by the temperature maintained during the fermentation process [30].

### 2.2. Assessment of Antioxidant Activity

To evaluate the antioxidant properties, two different assays were used to avoid possible irregularities in the performance of the tested extracts, given the differences in their principles of action. The antioxidant potential of all tested samples was evaluated by DPPH and ABTS assays. The DPPH assay is based on the hydrogen donating capacity to scavenge the DPPH radical. The DPPH radical in alcohol solution is purple in color. During the reaction, it accepts the electron donated by the antioxidant and loses its purple color, turning yellow in the presence of oxidants [31]. In the ABTS assay, the generation of the cation radical ABTS+ involves the direct generation of the green-blue chromophore ABTS in the reaction between ABTS and potassium persulfate. The radical formed during the reaction is blue-green in color. By reducing the cationic radical, antioxidants cause the color of the solution to fade, with the decrease in color intensity depending on the amount of antioxidants in the solution [32]. The measure of antioxidant activity is the value of IC50 parameter, which determines the concentration of antioxidant that causes a 50% decrease in the initial radical concentration. The antioxidant properties of the extracts and ferments were tested over a concentration range of 30 µg/mL to 3000 µg/mL. The IC_50_ results for DPPH and ABTS assays are shown in Table 3 and Table 4.

It was observed that both IC_50_ values determined for DPPH and ABTS assays differed for the extracts and their ferments. Analyzing the DPPH test, the lowest IC_50_ value indicating the best antioxidative properties was found for F20 (IC_50_ = 91.4 µg/mL ± 0.17) and for F10 (IC_50_ = 96.2 µg/mL ± 0.18) for *R. fruticosus* L. (in comparison, IC_50_ for the extract was 324.5 µg/mL ± 0.25 µg/mL). Additionally, ferments from *V. myrtillus* L. leaves showed high antioxidant properties. The value of IC_50_ was 118.3 µg/mL ± 0.18 µg/mL for F10 and 105.2 µg/mL ± 0.08 for F20 (for the extract value of IC_50_ = 494.7 µg/mL ± 0.31). These values indicate that kombucha ferments have stronger antioxidant properties than unfermented plants, but the time differences in values between fermentation times are not significant. Stronger free radical scavenging (lower IC_50_ values) was observed in the ABTS assay. However, the differences between the extract and the ferments are not significant.

Many authors have demonstrated the strong antioxidant properties of blueberry leaves due to the high content of phenolic acids and flavonoids, which are recognized free radical scavengers and inhibitors [9,33]. The antioxidant activity of a plant extract is not usually associated with a single phenolic compound. Several in vitro studies have shown that flavonoids, coumarins, phenolic acids, lignans, hydroxycinnamones and stilbenes have combined antioxidant activity. The antioxidant capacity of phenols is mainly due to their redox properties that allow them to act as reducers, hydrogen donors and singlet oxygen quenchers [34]. Moreover, fermented plant extracts have a documented high content of active substances. Jayabalan et al. have shown that total phenolic compounds, scavenging activity on DPPH radical, superoxide radical and inhibitory activity against hydroxyl radical-mediated linoleic acid were increased with an increase in fermentation time [30]. In addiction, Bhattacharya and Gachhui have shown that the antioxidant activity of kombucha tea is due to the presence of tea polyphenols and ascorbic acid. Kombucha exhibits higher antioxidant activity than unfermented tea, which may be due to the production of low molecular weight components and structural modification of tea polyphenols by enzymes produced by bacteria and yeast during fermentation. Kombucha exhibited increased free radical scavenging activity during fermentation. The extent of this activity depended on fermentation time, type of tea material and normal microbiota kombucha culture, which, in turn, determined the nature of their metabolites [35].

**Figure 2:**
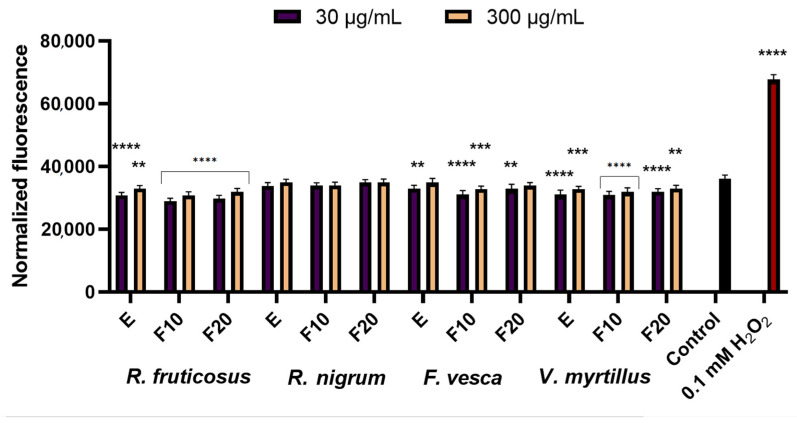
Effect of *R. fruticosus* L., *R. nigrum* L., *F. vesca* L., *V. myrtillus* L. extract and kombucha ferments (at concentrations of 30 µg/mL and 300 µg/mL) on the DCF fluorescence in fibroblasts (BJ). Data are the mean ± SD of three independent experiments, each consisting of three replicates per treatment group. **** *p* < 0.0001, *** *p* < 0.0010, ** *p* < 0.0020 versus the control.

**Figure 3:**
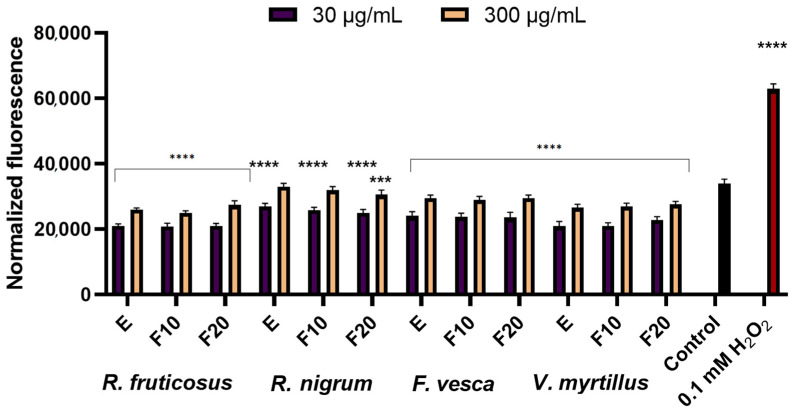
Effect of *R. fruticosus* L., *R. nigrum* L., *F. vesca* L., *V. myrtillus* L. extract and kombucha ferments (at concentrations of 30 µg/mL and 300 µg/mL) on the DCF fluorescence in keratinocytes (HaCaT). Data are the mean ± SD of three independent experiments, each consisting of three replicates per treatment group. **** *p* < 0.0001, *** *p* < 0.0004 versus the control.

In the next stage of the research, the ability of the berry leaf extracts and the obtained ferments to reduce intracellular production of reactive oxygen species (ROS) on keratinocytes (HaCaT) and fibroblast (BJ) cell lines was evaluated. Free radicals (reactive oxygen forms), generated in the skin cells, are one of the major factors inducing the skin ageing process. The study was performed using fluorogenic H2DCFDA dye. An H2DCFDA probe was used to detect redox imbalance after exposure of the test cells to berry leaves extracts and ferments, as it can react with several ROS, including hydroxyl radicals, hydrogen peroxide and peroxynitrite [36]. The analysis showed that the potential to reduce intracellular ROS production by kombucha extracts and ferments at the concentrations tested (30 µg/mL and 300 µg/mL) was demonstrated by both fibroblasts and keratinocytes (HaCaT), as normalized fluorescence values were lower than in control cells (cells cultured in medium without added extract or ferments) (Figure 2 and Figure 3). Additionally,0.1 mM H_2_O_2_ solution used as a positive test. A stronger potential to minimize oxidative stress in fibroblasts and HaCaT was demonstrated for extracts and ferments at a concentration of 30 μg/mL than at a concentration of 300 μg/mL. Extracts and ferments from *V. myrtillus* L. and *R. fruticosus* L. leaves were most effective in reducing ROS levels in keratinocyte cells. Additionally, a stronger ability to reduce intracellular oxidative stress was observed for HaCaT cells. Studies have also shown that both extracts and kombucha ferments have similarly strong antioxidant potential against skin cells.

Similar observations of reduced intracellular ROS production on cell lines by fermented plant extracts were reported by Ahn et al. [37]. They evaluated the antioxidant effects of Damdusi (a traditional fermented soybean product from Korea) extract by assessing it in non-cellular systems and cellular systems. In non-cellular systems, it showed scavenging activities on DPPH, hydroxyl and hydrogen peroxide radicals, and high chelating ability and reducing power. In cellular systems, it showed significant ROS scavenging ability, lipid peroxidation inhibition, and induced increases in levels of glutathione. Similar results were obtained by other researchers for fermented soybean extract [38,39], as well as for fermented garcinia beverage [40] and fermented dandelion beverage [41].

### 2.3. Cytotoxicity Assessment

In the next stage of the research, the influence of fermentation time on the viability of skin cells was assessed. The cytotoxicity of tested extracts was assessed with the use of HaCaT and BJ fibroblast cell lines using resazurin and neutral red method. Both types of cells were treated with various extract concentrations equaling 30 µg/mL and 300 µg/mL. The analyses were performed for four extracts obtained from plant leaves of varieties *R. fruticosus*, *R. nigrum*, *F. vesca*, *V. myrtillus*. It is well known that fruits are a rich source of biologically active compounds, which is confirmed by numerous studies. The leaves of these plants are often perceived as less valuable, often even as waste material; thus, the research on the usefulness of the leaves is much more numerous compared to the research on the fruit of the plant [42,43]. The research carried out as part of this study confirms that extracts obtained from leaves can be a valuable material, often exhibiting even more favorable properties than fruit extracts. There are several references in the literature confirming the same thesis. According to Gopalan, studies conducted with currant leaf extract have shown that it has stronger antioxidant properties compared to the extract of currant buds or fruit [44]. Another study claims that the anthocyanins present in blueberry leaves significantly reduce the negative impact of solar radiation on human fibroblasts, which may contribute to reducing the photoaging process of the skin and inhibiting collagen degradation [11,45].

For all extract samples analyzed within the presented paper, it was observed that the viability of the cells was above the control. On the other hand, the potential of the tested ferments differs depending on the concentration used and the analyzed cell line. However, with the time of fermentation (F10, F20), fluctuations in cell viability were observed. Based on the conducted analyses, it can be concluded that, in the vast majority of cases, the lower concentrations of ferments and the extract from the tested plants have a positive effect on the cell viability. It was observed that higher concentrations of leaf extracts inhibit the viability and metabolism of the tested cell lines. Long fermentation time (20 days) has a negative effect and reduces the cell viability. These data correspond with the thesis that long fermentation time may not be beneficial and may contribute to the accumulation of harmful products, including acids of organic acids, which might reach harmful levels for direct consumption [30,46].

**Figure 4:**
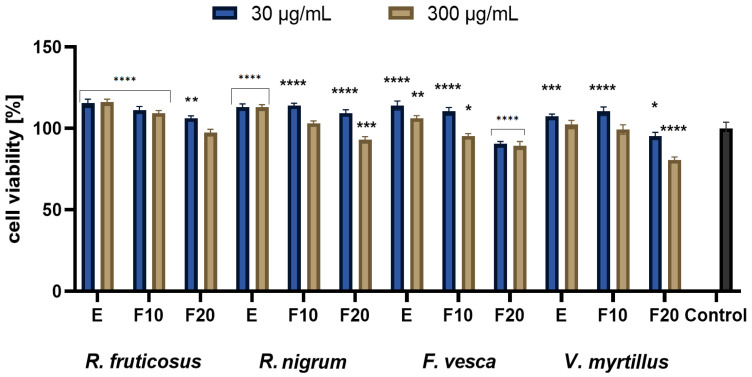
The reduction in resazurin after 24 h exposure to *R. fruticosus* L., *R. nigrum* L., *F. vesca* L., *V. myrtillus* L. extract and kombucha ferments (at concentrations of 30 and 300 µg/mL) in cultured fibroblasts (BJ). Data are the mean ± SD of three independent experiments, each of which consists of three replicates per treatment group., **** *p* < 0.0001, *** *p* < 0.0003, ** *p* < 0.0015, * *p* < 0.0035 versus the control (100%).

**Figure 5:**
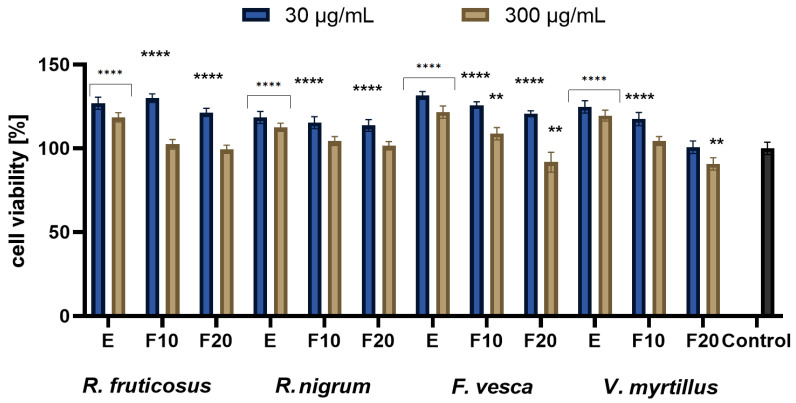
The reduction in resazurin after 24 h exposure to *R. fruticosus* L., *R. nigrum* L., *F. vesca* L., *V. myrtillus* L. extract and kombucha ferments (at concentrations of 30 and 300 µg/mL) in cultured keratinocytes (HaCaT). Data are the mean ± SD of three independent experiments, each of which consists of three replicates per treatment group. **** *p* < 0.0001, ** *p* < 0.0060 versus the control (100%).

The studies conducted with the Alamar Blue test showed that the greatest increase in proliferation was observed for extracts at a concentration of 30 µg/mL, where, in general, the highest values of cell viability were observed at the beginning of experiments, then decreasing with time lapse. A greater increase was seen with HaCaT compared to BJ. Performed analysis presented in Figure 4 showed that in case of concentration of 30 µg/mL differences between extracts were minimal at the beginning (E), only the *V. myrtillus* L. exhibited slightly lower cell viability compared to other extracts. For that concentration and F20, the highest values were observed for *R. nigrum* L. and the lowest for *F. vesca* L. An analogous comparison in the case of a concentration of 300 µg/mL shows that, for E, the highest values were observed for *R. fruticosus* and *R. nigrum*, whereas the value for *V. myrtillus* was slightly lower. For that concentration at F20, the highest values were observed for *R. fruticosus* and *R. nigrum*, whereas those for *V. myrtillus.* were the lowest. In another experiment, presented in Figure 5, the analysis comparing cell viability between extracts showed that, in the case of a concentration of 30 µg/mL and E, the highest value was measured for *F. vesca*, and the lowest for *R. nigrum*, whereas for F20, the highest value was observed for *R. fruticosus* and the lowest for *V. myrtillus.* For a concentration of 300 µg/mL and E, the highest value was measured for *F. vesca* and *V. myrtillus*, whereas the lowest was for *R. nigrum* L.; for F20, the highest value was observed for *R. fruticosus* and *R. nigrum* L., and the lowest equally for *F. vesca* and *V. myrtillus*. The studies conducted with the Neutral Red showed very similar general results to the one with the Alamar Blue. Again, the greatest increase in proliferation was observed for extracts at the concentration of 30 µg/mL with the highest values of cell viability at the beginning of experiments, which were then decreasing with time lapse. Additionally, again, a greater increase was seen with HaCaT compared to BJ.

**Figure 6:**
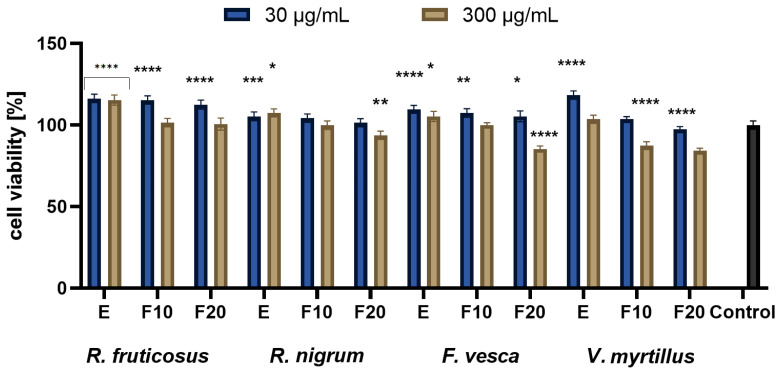
The effect of 24 h exposure of the *R. fruticosus* L., *R. nigrum* L., *F. vesca* L., *V. myrtillus* L. extract and kombucha ferments (at concentrations of 30 and 300 µg/mL) on Neutral Red Dye uptake in cultured fibroblasts (BJ). Data are the mean ± SD of three independent experiments, each of which consists of three replicates per treatment group. **** *p* < 0.0001, *** *p* < 0.0008, ** *p* < 0.0010, * *p* < 0.0036 versus the control (100%).

**Figure 7:**
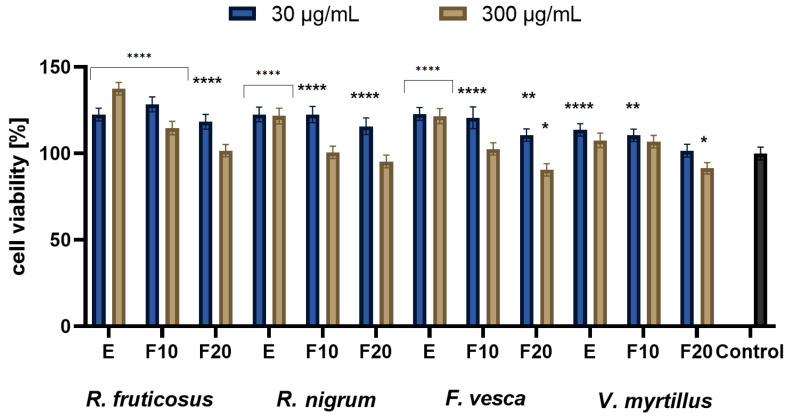
The effect of 24 h exposure of *R. fruticosus* L., *R. nigrum* L., *F. vesca* L., *V. myrtillus* L. extract and kombucha ferments (30 and 300 µg/mL) on Neutral Red Dye uptake in cultured keratinocytes (HaCaT). Data are the mean ± SD of three independent experiments, each of which consists of three replicates per treatment group. **** *p* < 0.0001, ** *p* < 0.0045, * *p* < 0.0330 versus the control (100%).

As presented in Figure 6, when comparing cell viability between extracts, in the case of a concentration of 30 µg/mL and E, the highest value was measured for *V. myrtillus*, whereas the lowest was for *R. nigrum*; for F20, the highest value was observed for *R. fruticosus* and the lowest for *V. myrtillus.* For a concentration of 300 µg/mL and E, the highest value was measured for *R. fruticosus*, whereas the lowest was equally for *V. myrtillus* and *F. vesca*; for F20, the highest value was observed for *R. fruticosus* and the lowest equally for *V. myrtillus* and *F. vesca.* The results of the last experiment are presented in Figure 7. A comparison of extracts showed that, in the case of a concentration of 30 µg/mL and E, the highest value was measured for *R. nigrum*, *F. vesca* and *R. fruticosus*, whereas the lowest was for *V. myrtillus* L. For F20, the highest value was observed for *R. fruticosus* and *R. nigrum*, whereas the lowest was for *V. myrtillus*. For a concentration of 300 µg/mL and E, the highest value was measured for *R. fruticosus*, and the lowest was for *V. myrtillus*; for F20, the highest value was observed for *R. fruticosus* and the lowest for *F. vesca* and *V. myrtillus.*.

Numerous studies in vitro, in vivo and clinical studies indicate that fruit is a rich source of bioactive compounds; various authors have tried to prove that leaves can also be a valuable source of extremely valuable and therapeutic compounds. The research carried out as part of this work and the cited literature data show that extracts obtained from the leaves of these plants, which are mainly seen as waste material, show even better properties than extracts from fruits [47]. Despite the many proven health benefits of SCOBY [48], there is still little research into the effects of kombucha on skin cells. The previous studies of Yerba Mate Kombucha ferments have shown positive effects on the viability of the cell lines tested—fibroblasts and keratinocytes [21]. The results obtained indicate that the compounds formed during the fermentation of Kombucha berry leaves may have a positive effect on skin cell viability, which is undoubtedly related to a wide range of biologically active compounds, whose presence was confirmed in the chromatographic analysis. Many studies have shown that black and green tea kombucha exhibits high or moderate cytotoxicity against cancer cells [49,50]. The main mechanisms of the antiproliferative and anticancer effects of polyphenols present in kombucha may be related to their antioxidant capacity, attributed to the radical scavenging mechanism [51]. Furthermore, these compounds may modulate various signaling pathways and proteins, involving markers of cellular proliferation, such as p53, p21 and ROS [52].

### 2.4. Assessment of Matrix Metallopeptidades Inhibition

**Figure 8:**
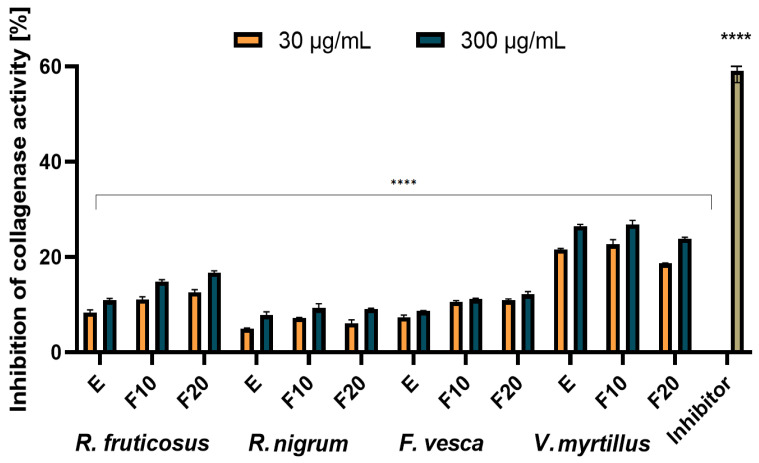
Collagenase inhibitory activity of *R. fruticosus* L., *R. nigrum* L., *F. vesca* L., *V. myrtillus* L. extract and kombucha ferments at concentrations of 30 and 300 µg/mL. Data are the mean of three independent experiments, each consisting of two replicates per treatment group. **** *p* < 0.0001 versus the control.

**Figure 9:**
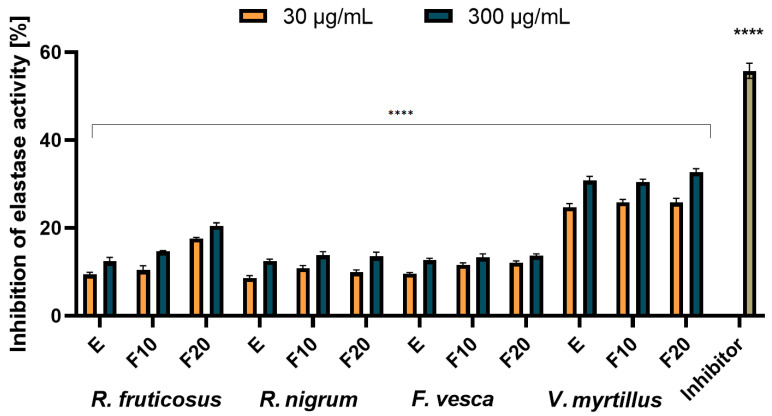
Elastase inhibitory activity of *R. fruticosus* L., *R. nigrum* L., *F. vesca* L., *V. myrtillus* L. extract and kombucha ferments at concentrations of 30 and 300 µg/mL. Data are the mean of three independent experiments, each consisting of two replicates per treatment group. **** *p* < 0.0001 versus the control.

Collagen and elastin are two major structural proteins that build human skin and are responsible for maintaining its elasticity and firmness [53]. With increasing age, the amount of these proteins in the skin decreases, which is one of the most important reasons for the skin aging process. Degradation of the skin collagen and elastin is caused by two matrix metallopeptidases, called collagenase and elastase. Their increased concentration in the skin is a natural process and occurs with age and may be accelerated by external factors such as diet, smoking, excessive exposure to the sun and UV radiation or prolonged oxidative stress caused by the lack or deficit of factors with antioxidant properties. A lot of substances that have been identified in plants have the ability to inhibit collagenase and elastase in the skin and prevent from collagen and elastin degradation [53,54,55,56]. The results of testing the ability of the analyzed extracts and ferments to inhibit collagenase and elastase are presented in Figure 8 and Figure 9.

It was shown that for both collagenase and elastase, the analysed ferments exhibited similar or slightly better inhibition properties of these enzymes compared to pure extracts. Time of the fermentation process did not have a significant impact on the results, and the ferments after ten and twenty days achieved comparable results. The strongest properties were observed for the extract and ferment obtained from leaves of *V. myrtillus*. For this plant, collagenase inhibition was noted on the level of around 20–22% (30 µg/mL) and 25–28% (300 µg/mL). The higher values of the analyzed parameter were noted in the case of elastase inhibition: 25–26% at the concentration of 30 µg/mL and around 30–32% at the concentration of 300 µg/mL. Ferments and extracts of *R. nigrum* and *F. vesca* are characterized by the lowest elastase and collagenase inhibition properties. At a concentration of 300 µg/mL, inhibition of collagenase by *R. nigrum* extract and ferments and *F. vesca* extract was observed at a level below 10% (6–8%), and for *R. vesca* ferment, at around 10%. The higher values were noted in the case of determining the analyzed parameter for elastase and an inhibition of around 15% was observed for both the extracts and the ferments.

The inhibition of elastase and collagenase by extracts obtained from leaves of the analyzed plants has not been studied so far. There are only a few studies in the literature on the inhibitory effect of blueberry and strawberry fruit extracts (obtained using water and ethanol as an extractant) on enzymes such as elastase, collagenase or tyrosinase [57,58,59]. It has been shown that anthocyanins and vitamin C, the main active substances of the fruits, are responsible for anti-collagenase and anti-elastase action. In the case of the analyzed leaf extracts and ferments the highest values of collagenase and elastase inhibitory action were observed for the *V. myrtillus* extract and ferments, and it was due to the fact that these samples contain the largest amount of identified active substances (expressed as the sum of all identified components in HPLC study). They are a rich source of chlorogenic acid and a derivative of quercetin, known for their strong antioxidant properties and capable of inhibiting the analyzed enzymes responsible for the aging process of the skin [53,55].

### 2.5. Influence of the Extracts and Ferments on the Skin Condition

Dry, damaged and inflexible skin is not only an aesthetic problem. The stratum corneum needs an adequate amount of water to fulfill its protective barrier function. Proper moisturizing of the skin also plays an important role in wound healing and skin regeneration as well as in delaying the skin aging processes. It is known that cosmetic preparations containing plant extracts have a positive effect on the skin, especially dry skin [60]. Fermented plant extracts have gained importance as a source of many active ingredients with a broad spectrum of activity. Due to the content of simple chemical compounds with low molecular weight, plant ferments are a source of bioavailable active substances, which are characterized by a high level of penetration into the deeper skin layers [61].

**Figure 10:**
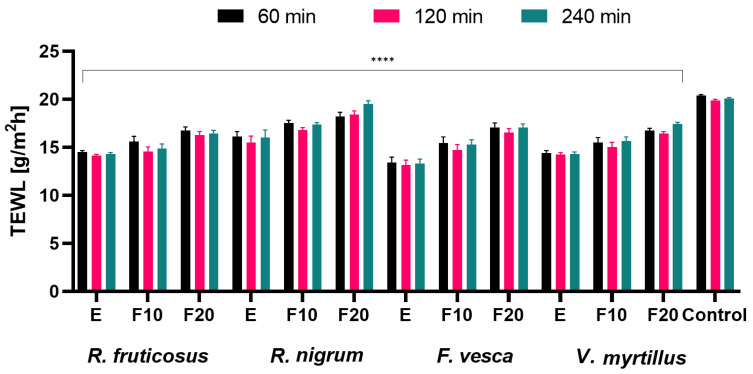
The influence of *R. fruticosus* L., *R. nigrum* L., *F. vesca* L., *V. myrtillus* L. extract and kombucha ferments (at concentration of 300 µg/mL) on transepidermal water loss (TEWL). Data are the mean ± SD of five independent measurements. **** *p* < 0.0001 versus the control.

**Figure 11:**
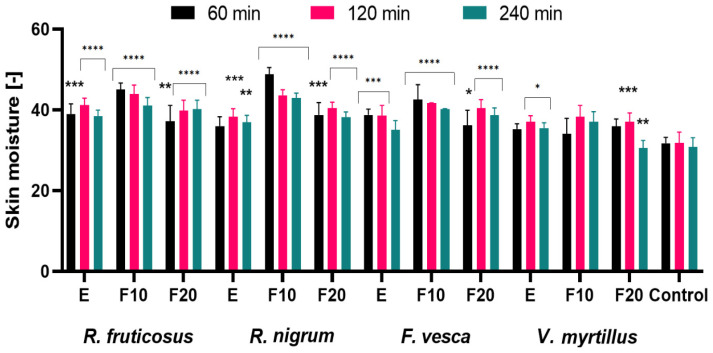
The influence of *R. fruticosus* L., *R. nigrum* L., *F. vesca* L., *V. myrtillus* L. extract and kombucha ferments (at concentration of 300 µg/mL) on skin hydration. Data are the mean ± SD of five independent measurements. **** *p* < 0.0001, *** *p* < 0.0008, ** *p* < 0.0080, * *p* < 0.050. versus the control.

For this purpose, hydration and transepidermal water loss (TEWL) analyses were carried out evaluating the effects of the tested extracts and their ferments on the skin. Measurements were made at three time intervals of 60, 120 and 240 min for an extract concentration of 300 µg/mL. The results are shown in Figure 10 and Figure 11.

Our study determined that all tested extracts and ferments exhibited a decrease in TEWL level, with *F. vesca* extract showing the most beneficial properties (compared to the control field, application of these samples to the skin surface resulted in a decrease in TEWL values by approximately 35% after 60, 120 and 240 min, respectively). Furthermore, the exposure time of the extracts and ferments on the skin showed no significant differences in decreasing TEWL level. Comparing the fermentation times of the tested samples, it should be concluded that for all tested plants, the extended fermentation time up to 20 days showed less beneficial preventing transepidermal leakage of water from the epidermis (compared to F10). It was also observed that the use of the tested plant extracts and ferments had a positive effect on skin hydration level. The most beneficial properties were observed for *R. nigrum* ferment after 20 days of fermentation. After 60 min from the application of the substance, the hydration level increased by 15% compared to the control field. With the passage of time, the trend in the level of hydration remained similar for all samples tested. The previously described HPLC analysis of berry leaf extracts and ferments showed a rich content of phenolic compounds and flavonoids, which, in addition to being well-known antioxidants, have a beneficial effect on the skin [62]. The study also showed that ferments after 10 days of fermentation showed the most beneficial effect on hydration. These changes can be explained by the fact that, as fermentation time increases, more acetic acid accumulates in the beverage and the content of simple sugars such as glucose and fructose decreases [63,64]. Simple sugars contain hydroxyl groups in their structure and are therefore valuable humectants and have a lower molecular weight and smaller particle size, which may affect their greater ability to penetrate deeper into the epidermis than complex substances, which act mainly at the skin surface [45]. Therefore, the fermentation time of kombucha plant extracts should be optimized in order to obtain the most beneficial cosmetic properties.

**Figure 12:**
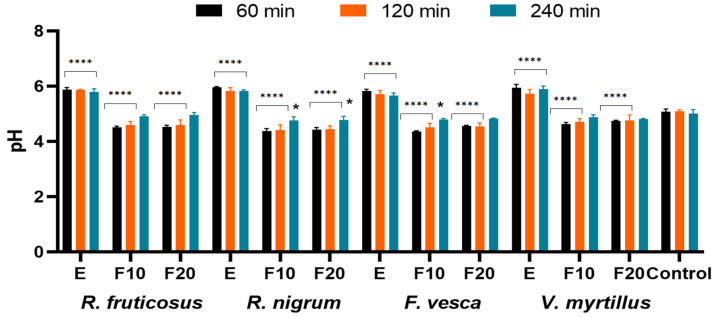
The influence of *R. fruticosus* L., *R. nigrum* L., *F. vesca* L., *V. myrtillus* L. extract and kombucha ferments (at concentration of 300 µg/mL) on skin pH. Data are the mean ± SD of five independent measurements. **** *p* < 0.0001, * *p* < 0.040. versus the control.

In the next stage of in vivo tests, the effect of the analyzed extracts and ferments on the skin pH was determined by conducting a skin pH meter test. The results are presented in Figure 12. Proper skin pH value is one of the most important factors influencing the skin condition and skin microbiota. Disturbance in the physiological pH of the skin, which occurs, among others, after the use of cleansing cosmetics (increasing the pH of the skin), may weaken the skin barrier function and inhibit the reconstruction of the skin microbiome and its regeneration [65,66,67,68,69,70,71]. The proper condition of the skin microbiome is extremely important in many skin diseases, such as acne, atopy or in the process of wound healing. Disturbing of the skin microbiome as a result of an increase in its pH may intensify pathological changes and impede wound healing [68,69,71]. That is why it is so important to use cosmetic products that do not cause skin pH growth after their application.

The results show that the analyzed plant extracts increase the pH value of the skin, which is still observed even after 240 min after applying them. During the study, no significant differences were observed between the extracts from the analyzed plants, and an increase in pH of about 0.5 pH units compared to the control was identified for all of them. After application on the skin of fermented extracts, a decrease in the skin pH was observed. After 60 min from the application of the ferments on the skin, the decrease in pH was about 0.5 pH units, and after 240 min, the pH value of the skin at the place of application of the ferments returned to the physiological state and did not differ from the value in the control field. Ferments from *R. fruticosus*, *R. nigrum* and *F. vesca* showed the strongest properties to lower the pH of the skin. There was no significant influence of fermentation time on the value of the analyzed parameter. The obtained results indicate that ferments can be valuable, new raw materials for the cosmetics industry. They are not only a rich source of active ingredients, such as antioxidants, but also have a positive effect on the pH of the skin, reducing it. Especially after using face and body cleansing cosmetics, an increase in skin pH is observed due to the surfactants contained in their formulations. It is very important to lower the pH to the physiological value in order to maintain the healthy and good condition of the skin and the proper condition of the skin microbiome. Ferments obtained from the analyzed plants added to cosmetics preparations, such as tonics or creams, can help the skin to return to its physiological pH.

## 3. Materials and Methods

### 3.1. Plant Material and Fermentation Procedure

The plant material was purchased from Dary Natury company—a Polish producer and distributor of herbs (Poland). The leaves of *R. nigrum* L., *V. myrtillus* L., *R. fruticosus* L. and *F. vesca* L. were collected on controlled and organic plantations. No chemical fertilizers or plant protection products were used in the cultivation. In addition, after obtaining the plant material, a preliminary selection was carried out, paying special attention to chemotaxonomic factors. Kombucha starter cultures were purchased from a commercial source from Poland. Prior to fermentation, Kombucha starter cultures were stored under aseptic conditions in a refrigerator (4 °C) and consisted of an acid broth and cellulose layer. Initially, an infusion of berry leaf extracts was prepared in a sterile beaker by mixing 15 g of leaves and 500 mL of hot distilled water (95 °C). Extracts were obtained by ultrasound-assisted extraction (UAE). UAE was performed according to the method described by Yang et al. [72] in an ultrasonic bath (Digital Ultrasonic Cleaner, Berlin, Germany) equipped with a time controller. Then, 50 g of sucrose (final concentration 10.0% *m*/*v*) was added to the extracts. The mixture thus prepared was stirred every few minutes with a glass chopstick until the solution was cooled (about 25 °C, cooling bath, cooling time 30–40 min). The resulting berry leaf infusion was then filtered twice through membrane filters into sterile glass beakers (1000 mL, height 18 cm, diameter 8 cm). Tea fungus (3 g) and Kombucha (50 mL) were added to the filtrate, and fermentation was carried out for 10 and 20 days (in separate beakers) at room temperature (approximately 25 °C). After completion of fermentation, the resulting Kombucha was filtered. Ferments obtained after 10 days were designated as F10 and after 20 days as F20.

### 3.2. Determination of Biologically Active Compounds

Main metabolites (phenolic acids and flavonoids) were using an ultra-high-performance liquid chromatography (UHPLC) Infnity Series II with a DAD detector and Agilent 6224 ESI/TOF mass detector (Agilent Technologies, Santa Clara, CA, USA). HPLC conditions were as follows: an RP18 reversed-phase column Titan (Supelco, Sigma-Aldrich, Burlington, MA, USA) (10 cm × 2.1 mm i.d., 1.9 μm particle size), a thermostat temperature of 30 °C and a flow rate of 0.2 mL/min. A mixture of water with 0.05% of formic acid (solvent A) and acetonitrile with 0.05% of formic acid (solvent B) was used as a mobile phase. The compounds were separated using gradient elution according to the program: 0–9 min from 98% A to 95% A (from 2% to 5% B), 9–24 min from 95% A to 92% A (from 5% to 8% B), 24–45 min from 92% A to 85% A (from 8% to 15% B) and 45–60 min from 85% A to 70% A (from 15% B to 30% B). Chromatograms were recorded from 200 to 400 nm. LC–MS analysis: the ion source operating parameters were as follows: drying gas temperature 325 °C, drying gas flow 5.1 min^−1^, nebulizer pressure 30 psi, capillary voltage 3500 V, fragmentator 170 V and skimmer 65 V. Ions were acquired in the range of 100 to 1050 *m*/*z*. MS identification was performed based on literature data and NIST database. Quantification was based on calibration curves obtained using methanol standard solutions of identified compounds. The content of the tested compounds is given in mg/mL. All standards were from Sigma-Aldrich (St. Louis, MO, USA).

### 3.3. Assessment of Antioxidant Activity

#### 3.3.1. DPPH Radical Scavenging Assay

The antioxidant activity of the tested ferments and extracts was carried out using the DPPH (1,1-diphenyl-2-picrylhydrazyl) radical [73]. The analyzed samples in concentrations: 30, 150, 300, 1500 and 3000 µg/mL were transferred to a 96-well plate (100 µL) and then 100 µL 4 mM methanol solution of DPPH was added and mixed well. Then, absorbance measurements were made every 5 min for half an hour at wavelength λ = 517 nm on a UV-VIS Filter Max λ = 5 spectrophotometer (Thermo Fisher Scientific, Waltham, MA, USA). Water with a DPPH solution was used as a control. Measurements were performed in three independent replications. The obtained results were used to calculate the percentage of DPPH radical scavenging using Equation (1):

(1) $$\%\;\mathrm{DPPH}\;\mathrm{scavenging}=\frac{\mathrm{Abs}\;\mathrm{control}-\mathrm{Abs}\;\mathrm{sample}}{\mathrm{Abs}\;\mathrm{control}}\times 100$$

where: As—absorbance of the sample; Ac—absorbance of the control sample.

From the obtained results the IC_50_ point was determined.

#### 3.3.2. ABTS+ Scavenging Assay

The second method of determining antioxidant properties was based on the use of ABTS solution [74]. For this purpose, the 7 mM ABTS solution and the 2.4 mM potassium persulfate solution were mixed in equal proportions and left for at least 14 h at room temperature. The resulting solution was then diluted in methanol until an absorbance of about 1.0 was obtained (λ = 734 nm). Next, 1 mL of testes samples (in concentrations: 30, 150, 300, 1500 and 3000 µg/mL) was mixed with 1 mL of ABTS solution, and the absorbance was measured at λ = 734 nm using UV/VIS spectrophotometer Aquamate Helion (Thermo Fisher Scientific, Waltham, MA, USA). Additionally, 1 mL of methanol mixed with 1 mL of ABTS was used as a control. The ABTS+ scavenging was calculated from Equation (2):

(2) $$\%\;\mathrm{of}\;\mathrm{ABTS}⦁+\mathrm{scavenging}\;=\frac{1-\mathrm{As}}{\mathrm{Ac}}\times 100$$

where: As—absorbance of the sample; Ac—absorbance of the control sample. Measurements were carried out in triplicate for each extract sample.

From the obtained results, the IC_50_ point was determined.

#### 3.3.3. Determination of Intracellular Levels of Reactive Oxygen Species (ROS)

In order to determine the antioxidant capacity of the tested ferments and extracts, their ability to intracellular production of reactive oxygen species was also assessed. For this purpose, a test was performed on human fibroblasts and keratinocytes using a fluorogenic dye H_2_DCFDA that enters the cell, where it is then converted into a non-fluorescent compound. In the presence of ROS, this compound is converted to fluorescent DCF. To determine the intracellular level of ROS in fibroblasts and keratinocytes, cells were seeded in 96-well plates and cultured in an incubator for 24 h. After this time medium was removed and replaced with 10 µM H_2_DCFDA (Sigma Aldrich, St. Louis, MO, USA) dissolved in serum free DMEM medium and cells were incubated for 45 min. Then, HaCaT and BJ cells were incubated with analyzed samples in the concentrations: 30 and 300 µg/mL. As a positive control, cells treated with 1 mM H_2_O_2_ were used, and as a control, cells untreated with the analyzed extracts were used. The fluorescence was measured every 90 min using a FilterMax F5 microplate reader (Thermo Fisher Scientific, Waltham, MA, USA) at a maximum excitation of 485 nm and emission spectra of 530 nm [75].

### 3.4. Cytotoxicity Analysis

#### 3.4.1. Cell Culture

Skin cells used in this study were grown in Dulbecco’s Modification of Eagle’s Medium (DMEM, Biological Industries, Cromwell, CO, USA) with the addition of sodium pyruvate, L-glutamine, 10% fetal bovine serum (Gibco, Waltham, MA, USA) and high glucose content (4.5 g/L). Medium was also enriched with 1% antibiotics (100 U/mL penicillin and 1000 µg/mL streptomycin, Gibco) to prevent microbial contamination. HaCaTs were obtained from CLS Cell Lines Service (CLS Cell Lines Service GmbH, Eppelheim, Germany) and BJs from the American Type Culture Collection (Manassas, VA, USA). Cells were grown in an incubator in a humidified atmosphere of 95% air and 5% carbon dioxide and at 37 °C. When the cells obtained the required confluence, the medium was removed, and cells were washed twice with sterile PBS (phosphate-buffered saline). After that, the cells were detached from the bottom of the culture flasks with trypsin and then were placed in fresh DMEM medium. In the next step, the cells were plated in 96-well flat bottom plates and incubated for at least 24 h. After attaching to the bottom of the plates, the cells were treated with extracts in concentrations: 30 and 300 µg/mL and incubated for 24 h. After this time, further analyses were carried out.

#### 3.4.2. Alamar Blue Assay

The first cytotoxicity test was Alamar Blue assay that is based on the use of resazurin solution. After incubating cells with analyzed samples, a resazurin solution was added to the wells at a concentration of 60 µM. Plates were placed in an incubator for 2 h at 37 °C. Then, fluorescence was measured at wavelength λ = 570 nm. Each sample was performed in three replications.

#### 3.4.3. Neutral Red Uptake Assay

The second cytotoxicity test was Neutral Red Uptake Assay. After incubating cells with analyzed samples, the neutral red dye at a concentration of 40 µg/mL was added to the wells. Plates were placed in an incubator for 2 h at 37 °C, then the neutral red dye was removed, and the cells were washed with PBS. After this, PBS was removed and 150 µL of decolorizing buffer was added and the absorbance measurements were performed at wavelength λ = 540 nm. Each sample was performed in three replications.

### 3.5. Assessment of Matrix Metallopeptidades Inhibition

#### 3.5.1. Determination of Anti-Collagenase Activity

The determination of anti-collagenase activity was carried out using a fluorometric kit (Abcam, Cambridge, UK, ab211108) in accordance with the instructions attached to the kit and with the procedure described by Nizioł-Łukaszewska et al. [76]. Analyzed extracts and ferments in concentration of 30 and 300 µg/mL were plated in a 96-well clear flat bottom plate. Collagenase (COL) was dissolved in a CAB (collagenase analysis buffer). Then, COL and CAB was added to the wells. Then, 80 mM 1,10-phenanthroline, which is a collagenase inhibitor, was mixed with collagenase and CAB, which was the inhibitor control sample. Enzyme control wells were prepared by mixing diluted COL with CAB. The CAB buffer was used as a background control. The samples were incubated for 15 min at room temperature. In the meantime, collagenase substrate was mixed with CAB, which was a reaction mixture. After incubation, the reaction mixture was added to the wells and mixed. Then, fluorescence was measured at excitation wavelength 490 nm and emission 520 nm. The measurement was performed in kinetic mode for 60 min at 37 °C. The ability to inhibit COL activity of analyzed extracts and ferments was calculated by Equation (3):

(3) $$\%\;\mathrm{relative}\;\mathrm{COL}\;\mathrm{inhibition}=\frac{\mathrm{enzyme}\;\mathrm{control}-\mathrm{sample}}{\mathrm{enzyme}\;\mathrm{control}}\times 100$$

#### 3.5.2. Determination of Anti-Elastase Activity

The determination of anti-elastase activity was carried out using a fluorometric kit (Abcam, ab118971) in accordance with the instructions attached to the kit and with the procedure described by Nizioł-Łukaszewska et al. [76]. Analyzed extracts and ferments in concentrations of 30 and 300 µg/mL were plated in a 96-well clear flat bottom plate. First, neutrophil elastase (NE) enzyme solutions, NE substrate and an inhibitor control (SPCK) were prepared according to the instructions. NE solution was diluted and added to the wells, and then the inhibitor control and the enzyme control (Assay Buffer) were added to subsequent wells. The samples were mixed and incubated for 5 min, at 37 °C. Assay Buffer was mixed with NE substrate, which was a reaction mixture. After incubation, the reaction mixture was added to the wells and mixed. Fluorescence was measured immediately at excitation wavelength λ = 400 nm and emission λ = 505 nm using a microplate reader (FilterMax F5, Thermo Fisher Scientific, Waltham, MA, USA). The ability to inhibit NE activity of the analyzed samples was calculated from Equation (4):

(4) $$\%\;\mathrm{relative}\;\mathrm{NE}\;\mathrm{activity}=\frac{\Delta \mathrm{RFU}\;\mathrm{test}\;\mathrm{inhibitor}}{\Delta \mathrm{RFU}\;\mathrm{enzyme}\;\mathrm{control}}\times 100$$

### 3.6. Measurement of Transepidermal Water Loss (TEWL), Skin Hydration and Skin pH

Measurements of TEWL, skin hydration and skin pH were performed using the TEWAmeter TM 300, Skin pH-meter PH905 and Corneometer CM 825 probe connected to the MPA adapter (Courage + Khazaka Electronic, Cologne, Germany). The study was conducted on 5 volunteers. Areas (measuring 2 × 2 cm) were marked on the skin of each volunteer forearm. Then, 20 µL of each sample (at a concentration of 300 µg/mL) was applied in the areas. One field (control field) was not treated with any sample. The samples were gently spread over each field and left to dry. After 60, 120 and 240 min, the level of hydration, pH and TEWL were measured. The final result was the arithmetic mean (from each volunteer) of 5 independent measurements (skin hydration and pH) and 20 measurements (TEWL).

### 3.7. Statistical Analysis

Values of different parameters were expressed as the mean ± standard deviation (SD). The two-way analysis of variance (ANOVA) and Bonferroni post-test between groups were performed at the *p*-value level of <0.05 to evaluate the significance of differences between values. Statistical analysis was performed using GraphPad Prism 8.4.3 (GraphPad Software, Inc., San Diego, CA, USA).

## 4. Conclusions

Numerous scientific studies point to the health-promoting properties of kombucha fermented with black or green tea. Furthermore, there is emerging work indicating that other substrates can be used for kombucha fermentation. HPLC/DAD/ESI-MS revealed a significant content of biologically active compounds, the values of which correlate with their antioxidant potential. In addition, both extracts and ferments have been shown to have positive effects on the viability and metabolism of skin cells—fibroblasts and keratinocytes. Berry leaf kombucha ferments have also been shown to contribute to the inhibition of collagenase and elastase activity having a great impact on the healthy appearance of the skin. The study also found that all the extracts and ferments tested showed a reduction in TEWL level and an increase in skin hydration level and have a positive effect on the skin’s pH, lowering it. There are differences in the context of biological activity, comparing ferments after 10 and 20 days of fermentation. The extracts after 20 days of fermentation showed significant antioxidant properties. However, the evaluation of skin cell cytotoxicity showed the most favorable effect after 10 days of fermentation. As fermentation time increased, cell viability decreased. The results obtained indicate that the ferments, in addition to probiotic activity, supporting beneficial microorganisms inhabiting human skin, may also be a valuable ingredient present in pharmaceutical and cosmetic products.

## Figures and Tables

**Table 1:** Bioactive compounds detected using UHPLC/DAD/ESI–MS in *R. fruticosus* L., *R. nigrum* L., *F. vesca* L., *V. myrtillus* L. An x indicates the occurrence of the detected compounds in the tested plant.

Molecular Formula	Name of Compound	*R. fruticosus*	*R. nigrum*	*F. vesca*	*V. myrtillus*
C_7_H_6_O_5_	Gallic acid	x	x	x	x
C_7_H_6_O_4_	Protocatechuic acid		x		x
C_16_H_18_O_9_	Neochlorgenic acid	x			
C_15_H_18_O_9_	Caffeoyl hexoside (I)	x			
C_15_H_18_O_9_	Caffeoyl hexoside (II)	x			
C_15_H_18_O_9_	Caffeoyl hexoside (III)	x			
C_16_H_18_O_9_	Chlorogenic acid	x	x	x	x
C_16_H_18_O_9_	Cryptochlorogenic acid		x		x
C_9_H_8_O_3_	*p*-coumaric acid		x		
C_16_H_18_O_8_	*p*-coumaroylquinic acid				x
C_19_H_14_O_12_	Ellagic acid pentoside	x		x	
C_25_H_28_O_13_	*p*-Coumaroyl monotropein				x
C_14_H_6_O_8_	Ellagic acid	x		x	
C_27_H_30_O_16_	Quercetin rhamnoside 3-*O*-glucoside		x		
C_27_H_30_O_16_	Rutin	x	x	x	
C_21_H_20_O_12_	Quercetin-3-*O*-galactoside				x
C_21_H_18_O_13_	Quercetin-3-*O*-glucuronide	x		x	x
C_27_H_27_O_16_	Quercetin hydroxymethyl glutaroyl hexoside			x	
C_21_H_18_O_12_	Luteolin 3-*O*-glucoronide	x			
C_27_H_29_O_15_	Kaempferol-3-*O*-rutinoside	x		x	
C_24_H_22_O_15_	Quercetin malonyl glucoside		x		
C_21_H_20_O_11_	Kaempferol glucoside		x		
C_21_H_17_O_12_	Kaempferol glucuronide			x	
C_21_H_18_O_12_	Kaempferol-3-Oglucoronide	x		x	
C_21_H_17_O_12_	Kaempferol hexuronide			x	x
C_21_H_18_O_12_	Dimethylellagic acid pentoside			x	
C_21_H_18_O_11_	Apigenin-3-*O*-glucoronide	x			
C_24_H_21_O_14_	Quercetin malonyl rhamnoside		x		

**Table 2:** UHPLC/DAD/ESI-MS quantitative analysis of *R. fruticosus* L., *R. nigrum* L., *F. vesca* L., *V. myrtillus* L. water extracts and kombucha ferments. Values are means ± standard deviation (SD) of triplicate.

Analyzed Plant	Name of Compound	Content (µg/mL)		
		Extract	F10 (10 Days)	F20 (20 Days)
*Rubus fruticosus L.*	Gallic acid	3.99 ± 0.12	7.68 ± 0.06	6.04 ± 0.07
	Neochlorgenic acid	1.05 ± 0.02	2.34 ± 0.00	2.77 ± 0.02
	Caffeoyl hexoside (I)	3.60 ± 0.01	7.13 ± 0.02	9.65 ± 0.10
	Caffeoyl hexoside (II)	6.58 ± 0.03	5.52 ± 0.01	4.45 ± 0.01
	Caffeoyl hexoside (III)	3.13 ± 0.03	5.82 ± 0.04	5.17 ± 0.02
	Chlorogenic acid	3.18 ± 0.01	5.84 ± 0.03	5.23 ± 0.01
	Ellagic acid pentoside	8.24 ± 0.42	23.27 ± 1.12	27.49 ± 0.65
	Ellagic acid	19.57 ± 0.86	80.87 ± 2.23	87.88 ± 0.42
	Rutoside	0.03 ± 0.00	2.23 ± 0.15	3.34 ± 0.09
	Quercetin glucuronide	7.12 ± 0.11	14.36 ± 0.21	21.68 ± 0.68
	Luteolin-3-*O*-glucoronide	6.12 ± 0.21	7.32 ± 0.07	8.89 ± 0.10
	Kaempferol-3-*O*-rutinoside	2.53 ± 0.01	4.54 ± 0.01	6.74 ± 0.04
	Kaempferol-3-*O*-glucoronide	6.86 ± 0.14	6.39 ± 0.13	5.94 ± 0.04
	Apigenin-3-*O*-glucoronide	-	12.38 ± 0.15	24.30 ± 1.14
	Quercetin glucoside	0.22 ± 0.02	2.86 ± 0.14	6.22 ± 0.13
*Ribes nigrum L.*	Gallic acid	1.68 ± 0.01	6.55 ± 0.02	5.22 ± 0.02
	Protocatechuic acid	6.56 ± 0.13	6.59 ± 0.01	6.54 ± 0.13
	Chlorogenic acid	8.84 ± 0.06	22.23 ± 0.57	21.22 ± 0.34
	Cryptochlorogenic acid	10.19 ± 0.54	11.68 ± 0.48	10.82 ± 0.30
	p-coumaric acid	6.03 ± 0.05	7.59 ± 0.07	7.46 ± 0.25
	Rutin	0.92 ± 0.01	25.13 ± 0.30	26.38 ± 0.28
	Quercetin 3-glucoside	3.82 ± 0.03	38.15 ± 0.12	38.46 ± 0.94
	Quercetin malonyl glucoside	11.95 ± 0.12	24.93 ± 0.17	23.63 ± 0.03
	Kaempferol glucoside	1.43 ± 0.08	20.75 ± 0.61	21.85 ± 0.06
	Quercetin malonyl rhamnoside	5.21 ± 0.06	7.99 ± 0.06	7.11 ± 0.15
*Fragaria vesca L.*	Gallic acid	1.38 ± 0.05	15.85 ± 0.06	15.51 ± 0.47
	Chlorogenic acid	-	4.72 ± 0.01	6.23 ± 0.05
	Elagic acid	9.97 ± 0.90	32.65 ± 0.78	75.58 ± 0.53
	Dimethylellagic acid pentoside	10.73 ± 0.28	10.01 ± 0.42	9.21 ± 0.53
	Elagic acid pentozyd	3.28 ± 0.13	21.68 ± 0.38	77.72 ± 1.12
	Rutoside	-	2.76 ± 0.18	4.23 ± 0.09
	Quercetin glucuronide	63.30 ± 0.00	56.37 ± 0.24	72.07 ± 0.05
	Quercetin hydroxymethylglutaroyl hexoside	12.00 ± 0.06	83.28 ± 0.26	161.73 ± 0.57
	Kaempferol coumaroyl hexoside	46.59 ± 0.74	21.51 ± 0.30	-
	Kaempferolglucuronide	65.78 ± 1.59	111.60 ± 3.10	177.44 ± 1.23
*Vaccinum myrtillus L.*	Gallic acid	-	5.03 ± 0.14	2.70 ± 0.15
	Protocatechuic acid	4.23 ± 0.09	5.32 ± 0.12	3.06 ± 0.15
	Chlorogenic acid	923.25 ± 1.43	1362.91 ± 8.88	1015.54 ± 1.83
	Cryptochlorogenic acid	54.94 ± 1.45	75.80 ± 0.65	61.51 ± 1.69
	*p*-coumaroylquinic acid II	5.40 ± 0.03	8.09 ± 0.10	6.80 ± 0.24
	*p*-Coumaroyl monotropein	11.27 ± 0.55	17.42 ± 0.28	13.57 ± 0.38
	*p*-Coumaroyl diacetylhexoside	26.68 ± 0.47	41.53 ± 1.02	31.20 ± 0.53
	*p*-Coumaroyl malonylhexoside I	5.83 ± 0.26	8.70 ± 0.14	6.67 ± 0.22
	*p*-Coumaroyl malonylhexoside II	30.67 ± 0.23	49.19 ± 0.24	40.18 ± 0.51
	Quercetin galactoside	11.90 ± 0.08	27.12 ± 1.33	17.75 ± 0.32
	Quercetin hexuronide	106.22 ± 0.23	172.68 ± 2.86	116.64 ± 1.06
	Kaempferol glucuronide	15.42 ± 0.37	23.49 ± 0.34	18.44 ± 0.34

**Table 3:** Values of IC_50_ of DPPH radical scavenging for *R. fruticosus* L., *R. nigrum* L., *F. vesca* L., *V. myrtillus* L. extract and kombucha ferments after 20 min of exposure. Values are means ± standard deviation (SD) of triplicate.

	Extract Plant	Ferment 10 Days	Ferment 20 Days
Type of Analyzed Plant	IC_50_ (µg/mL)		
*Rubus fruticosus* L.	324.5 ± 0.25	96.2 ± 0.18	91.4 ± 0.17
*Ribes nigrum* L.	1215.7 ± 0.36	278.3 ± 0.17	351.8 ± 0.37
*Fragaria vesca* L.	1172.5 ±0.13	121.3 ± 0.02	114.7 ± 0.16
*Vaccinum myrtillus* L.	494.7 ± 0.31	118.3 ± 0.18	105.2 ± 0.08

**Table 4:** Values of IC_50_ of ABTS+ radical scavenging for *R. fruticosus* L., *R. nigrum* L., *F. vesca* L., *V. myrtillus* L. extract and kombucha ferments. Values are mean ± standard deviation (SD) of triplicate.

	Extract Plant	Ferment 10 Days	Ferment 20 Days
Type of Analyzed Plant	IC_50_ (µg/mL)		
*Rubus fruticosus* L.	92.3 ± 0.04	89.4 ± 0.08	90.4 ± 0.12
*Ribes nigrum* L.	97.9 ± 0.06	94.3 ± 0.15	95.6 ± 0.21
*Fragaria vesca* L.	97.3 ± 0.08	94.3 ± 0.14	93.7 ± 0.19
*Vaccinum myrtillus* L.	92.7 ± 0.21	93.5 ± 0.10	93.1 ± 0.13

## Data Availability

Data are contained within the article.

## Supplementary Materials

The following are available online at https://www.mdpi.com/article/10.3390/molecules27072345/s1, Table S1: Characterization of metabolites identified in *Rubus fruticosus* extract by HPLC-MS in negative ion mode [24,77,78]; Table S2: Characterization of metabolites identified in *Ribes nigrum.* extract by HPLC-MS in negative ion mode [26,79]; Table S3: Characterization of metabolites identified in *Fragaria vesca*. extract by HPLC-MS in negative ion mode [27,80]; Table S4: Characterization of metabolites identified in *Vaccinum myrtillus*. extract by HPLC-MS in negative ion mode [25,78].

## Sample Availability

Samples of the compounds used and the plants extracts tested are available from the authors.
