# Supplementary material for: Evaluation of Cosmetic and Dermatological Properties of Kombucha-Fermented Berry Leaf Extracts Considered to Be By-Products
Source: Molecules. 2022 Apr 6;27(7):2345. doi: 10.3390/molecules27072345 (PMC9000515; doi:10.3390/molecules27072345)
Supplement: Supplementary file 1 [file molecules-27-02345-s001.zip › molecules-1655178-supplementary.pdf]

molecules

Supplementary material

Evaluation of cosmetic and dermatological properties of kombucha-fermented berry leaf extracts considered as by-products

Aleksandra Ziemlewska [1] *, Zofia Nizioł-Łukaszewska [1], Martyna Zagórska-Dziok [1], Tomasz Bujak [1], Magdalena Wójciak [2] and Ireneusz Sowa [2]

[1] Department of Technology of Cosmetic and Pharmaceutical Products, Medical College, University of Information Technology and Management in Rzeszow, Sucharskiego 2, 35-225 Rzeszow, Poland;
aziemlewska@wsiz.edu.pl (A.Z.); zniziol@wsiz.edu.pl (Z.N.-Ł.); mzagorska@wsiz.edu.pl (M.Z.-D.); tbujak@wsiz.edu.pl (T.B.)
[2] Department of Analytical Chemistry, Medical University of Lublin, Aleje Raclawickie 1, 20-059 Lublin, Poland; magdalenawojciak@umlub.pl (M.W.); i.sowa@umlub.pl (I.S.)
* Correspondence: aziemlewska@wsiz.edu.pl (A.Z.)

Table S1. Characterization of metabolites identified in *Rubus fruticosus* extract by HPLC-MS in negative ion mode

Rt (min.)	m/z-H	Error (ppm)	Fragments	Formula	Compound	Identification
4.82	169.0146	2.08	125	$C_7H_6O_5$	gallic acid	[1] (std)
12.02	353.0870	-2.28	179, 191	$C_{16}H_{18}O_9$	neochlorgenic acid	[2] (std)
17.13	341.0861	-4.99	179	$C_{15}H_{18}O_9$	caffeoyl hexoside (I)	[2]*
17.58	341.0875	-0.89	179	$C_{15}H_{18}O_9$	caffeoyl hexoside (II)	[2]*
19.75	341.0869	-2.65	179	$C_{15}H_{18}O_9$	caffeoyl hexoside (III)	[2]*
22.52	353. 0870	-2.28	179, 191	$C_{16}H_{18}O_9$	chlorogenic acid	[2] (std)
46.35	609.1460	-0.18	301	$C_{27}H_{30}O_{16}$	rutoside	[2] (std)
48.12	433.0417	1.04	301	$C_{19}H_{14}O_{12}$	ellagic acid pentoside	[1-3]**
48.97	300.9991	0.36		$C_{14}H_6O_8$	ellagic acid	[1-3] (std)
49.96	477.0678	0.7	301	$C_{21}H_{18}O_{13}$	quercetin-3-O-glucuronide	[1-3] (std)
51.11	461.0720	-1.19	285	$C_{21}H_{18}O_{12}$	luteolin 3-O-glucoronide	[2]***
51.59	593.154	4.72	285	$C_{27}H_{30}O_{15}$	kaempferol-3-O-rutinoside	[2]****
54.04	461.0720	-1.19	285	$C_{21}H_{18}O_{12}$	kaempferol -3-O-glucoronide	[2]****
54.67	445.0770	-1.42	269	$C_{21}H_{18}O_{11}$	apigenin-3-O-glucoronide	[2]*****

*Quantification was based on calibration curve for caffeic acid, ** Quantification was based on calibration curve for ellagic acid, *** Quantification was based on calibration curve for luteolin glucoside, **** Quantification was based on calibration curve for kaempferol glucoside, ***** Quantification was based on calibration curve for apigenin glucoside

Table S2. Characterization of metabolites identified in *Ribes nigrum* .extract by HPLC-MS in negative ion mode

Rt (min.)	m/z-H	Error (ppm)	Fragments	Formula	Compound	Identification
5.01	169.0135	-4.39	125	$C_7H_6O_5$	gallic acid	[4] (std)
8.95	153.0190	-2.16	127	$C_7H_6O_4$	protocatechuic acid	standard
21.84	353.0867	-3.12	179, 191	$C_{16}H_{18}O_9$	chlorogenic acid	[4] (std)
22.44	353.0863	-4.25	179, 191	$C_{16}H_{18}O_9$	cryptochlorogenic acid	[5] (std)
31.85	163.0405	2.64		$C_9H_8O_3$	p-coumaric acid	[5] (std)
46.34	609.1465	0.64	301	$C_{27}H_{30}O_{16}$	rutoside	[4,5] (std)
50.34	463.0900	3.88	301	$C_{21}H_{20}O_{12}$	quercetin 3-O-glucoside	[4] (std)
52.89	549.0889	0.56	301	$C_{24}H_{22}O_{15}$	quercetin malonyl glucoside	[4,5]*
54.22	447.0936	0.7	285	$C_{21}H_{20}O_{11}$	kaempferol glucoside	[4] (std)
56.37	532.0852	-1.23	301	$C_{24}H_{21}O_{14}$	quercetin malonyl rhamnoside	[4]*

*Quantification was based on calibration curve for rutoside

Table S3. Characterization of metabolites identified in *Fragaria vesca*.extract by HPLC-MS in negative ion mode

Rt (min.)	m/z-H	Error (ppm)	Fragments	Formula	Compound	Identification
4.97	169.0139	-2.04	125	$C_7H_6O_5$	gallic acid	[6] (std)
22.01	353.0872	-1.71	179, 191	$C_{16}H_{18}O_9$	chlorogenic acid	[6,7] (std)
45.66	623.1270	2.61	285	$C_{27}H_{28}O_{17}$	kaempferol hexoside glucuronide	[6,7]*
46.67	609.1460	-0.18	301	$C_{27}H_{30}O_{16}$	rutoside	[6,7] (std)
48.05	433.0413	0.12	301	$C_{19}H_{14}O_{12}$	ellagic acid pentoside	[6,7]**
49.41	300.9982	-2.62		$C_{14}H_6O_8$	ellagic acid	[6,7] (std)
49.48	477.0676	0.28	301	$C_{21}H_{18}O_{13}$	quercetin glucuronide	[6,7] (std)
50.33	607.1311	1.06	301	$C_{27}H_{28}O_{16}$	quercetin hydroxymethylglutaroyl hexoside	[6,7]***
51.50	593.1296	-0.78	285	$C_{30}H_{26}O_{13}$	kaempferol coumaroyl hexoside	[6,7]*
54.32	461.0746	4.44	285	$C_{21}H_{18}O_{12}$	kaempferolglucuronide	[6,7]*
55.01	461.0720	-1.19	315	$C_{21}H_{18}O_{12}$	dimethyl ellagic acid pentoside	[6,7]**

*Quantification was based on calibration curve for kaempferol glucoside, ** Quantification was based on calibration curve for ellagic acid, ***Quantification was based on calibration curve for quercetin glucoside,

Table S4. Characterization of metabolites identified in *Vaccinum myrtillus* .extract by HPLC-MS in negative ion mode

Rt (min.)	m/z-H	Error (ppm)	Frag-ments	Formula	Compound	Identification
4.68	169.0144	0.9	125	$C_7H_6O_5$	gallic acid	standard
8.93	153.0197	2.39	127	$C_7H_6O_4$	protocatechuic acid	[8] (std)
21.88	353.0891	3.66	179, 191	$C_{16}H_{18}O_9$	chlorogenic acid	[3] (std)
22.42	353.0888	2.81	179, 191	$C_{16}H_{18}O_9$	cryptochlorogenic acid	[3] (std)
35,40	337.0934	1.51	191	$C_{16}H_{18}O_8$	p-coumaroylquinic acid II	[3]*
47.72	535.1461	0.72	311,371	$C_{25}H_{28}O_{13}$	p-Coumaroyl monotropein	[8]*
49.22	463.0889	1.51	301	$C_{21}H_{20}O_{12}$	quercetin-3-O-galactoside	[3] (std)
49.63	477.0669	-1.18	301	$C_{21}H_{18}O_{13}$	quercetin glucuronide	[3] (std)
51.93	409.1149	2.14	163	$C_{19}H_{22}O_{10}$	p-Coumaroyl diacetylhexoside	[8]*
53.92	461.0719	-1.41	285	$C_{21}H_{18}O_{12}$	kaempferol glucuronide	[3]**
55.43	411.1680	4.72	163	$C_{20}H_{28}O_9$	p-Coumaroyl malonylhexoside I	[8]*
56.63	411.1677	3.99	163	$C_{20}H_{28}O_9$	p-Coumaroyl malonylhexoside II	[8]*

*Quantification was based on calibration curve for p-coumaric acid, ** Quantification was based on calibration curve for kaempferol glucoside

References

1. Mertz, C.; Cheynier, V.; Günata, Z.; Brat, P. Analysis of Phenolic Compounds in Two Blackberry Species (Rubus glaucus and Rubus adenotrichus) by High-Performance Liquid Chromatography with Diode Array Detection and Electrospray Ion Trap Mass Spectrometry. *J. Agric. Food Chem.* **2007**, 55, 8616–8624.
2. Oszmiański, J.; Wojdyło, A.; Nowicka, P.; Teleszko, M.; Cebulak, T.; Wolanin, M.; Determination of Phenolic Compounds and Antioxidant Activity in Leaves from Wild Rubus L. Species. *Molecules* **2015**, 20, 4951-4966.
3. Oszmianski, J.; Wojdyło, A.; Gorzelany, J.; Kapusta, I. Identification and Characterization of Low Molecular Weight Polyphenols in Berry Leaf Extracts by HPLC-DAD and LC-ESI/MS. *J. Agric. Food Chem.* **2011**, 59, 12830–12835.
4. D'Urso, G.; Montoro, P.; Piacente, S. Detection and comparison of phenolic compounds in different extracts of black currant leaves by liquid chromatography coupled with high-resolution ESI-LTQ-Orbitrap MS and high-sensitivity ESI-Qtrap MS. *J. Pharm. Biomed. Anal.* **2020**,179, 112926.
5. Charpentier, T.; Boisard, S., Le Ray, A.M.; Bréard, D.; Chabrier, A.; Esselin, H.; Guilet, D.; Ripoll C.; Richomme, P. A Descriptive Chemical Composition of Concentrated Bud Macerates through an Optimized SPE-HPLC-UV-MS2 Method—Application to Alnus glutinosa, Ribes nigrum, Rosa canina, Rosmarinus officinalis and Tilia tomentosa. *Plants* **2022**, 11, 144.
6. D'Urso, G.; Maldini, M.; Pintore G.; d'Aquino, L.; Montoro, P.; Pizza, C. Characterisation of Fragaria vesca fruit from Italy following a metabolomics approach through integrated mass spectrometry. Techniques. *LWT - Food Sci Technol.* **2016,** 74, 387-395.
7. D'Urso, G.; Pizza, C.; Piacente, S.; Montoro, P. Combination of LC–MS based metabolomics and antioxidant activity for evaluation of bioactive compounds in Fragaria vesca leaves from Italy. *J. Pharm. Biomed. Anal.* **2018**, 150, 233–240.
8. Bujor, O.C.; Le Bourvellec, C.; Volf, I.; Popa, V. I.; Dufour, C. Seasonal variations of the phenolic constituents in bilberry (Vaccinium myrtillus L.) leaves, stems and fruits, and their antioxidant activity. *Food Chemi*. **2016**, 213, 58–68.
